# Metal–Organic‐Framework‐Derived Dual Metal‐ and Nitrogen‐Doped Carbon as Efficient and Robust Oxygen Reduction Reaction Catalysts for Microbial Fuel Cells

**DOI:** 10.1002/advs.201500265

**Published:** 2015-12-03

**Authors:** Haolin Tang, Shichang Cai, Shilei Xie, Zhengbang Wang, Yexiang Tong, Mu Pan, Xihong Lu

**Affiliations:** ^1^ State Key Laboratory of Advanced Technology for Materials Synthesis and ProcessingWuhan University of TechnologyWuhan430070P. R. China; ^2^ MOE of the Key Laboratory of Bioinorganic and Synthetic ChemistrySchool of Chemistry and Chemical EngineeringSun Yat‐Sen UniversityGuangzhou510275P. R. China; ^3^ Institut für Funktionelle Grenzflächen (IFG)Karlsruher Institut für TechnologieHermann‐von‐Helmholtz‐Platz 176344Eggenstein‐LeopoldshafenGermany

**Keywords:** dual metal doping, metal organic framework, microbial fuel cells, oxygen reduction reaction, porous carbon

## Abstract

**A new class of dual metal and N doped carbon catalysts with well‐defined** porous structure derived from metal–organic frameworks (MOFs) has been developed as a high‐performance electrocatalyst for oxygen reduction reaction (ORR). Furthermore, the microbial fuel cell (MFC) device based on the as‐prepared Ni/Co and N codoped carbon as air cathode catalyst achieves a maximum power density of 4335.6 mW m^−2^ and excellent durability.

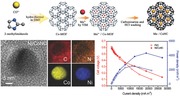

Energy shortage and environmental pollution are the most pressing issues that have to be addressed in order to realize the sustainable development of human society. Microbial fuel cells (MFCs) hold great potential to meet both challenges because they could decompose organic waste through biological oxidation and produce electrical energy simultaneously.^1^ Like a traditional fuel cell, MFC is composed of an anode for fuel oxidation, an ion exchange membrane, and a cathode that consumes electrons for oxidant reduction.^2^ Organic matters such as glucose are generally employed as the fuel for MFC at the anode and oxygen is the most commonly used oxidant at the cathode. MFCs have witnessed rapid development over the last few years owing to their attractive benefits. However, there are still technical hurdles that prevent their widespread application for renewable energy production. One of the major challenges toward an applicable MFC is the high cost of the MFC system. The conventional cathode catalyst, which functions as the oxygen reduction reaction (ORR) catalyst, is based on noble metal such as platinum and accounts for approximately 50% of the total cost.^3^, ^4^ Thus, replacing the noble metal based cathode catalyst with the low‐cost catalyst is a favorable approach to reduce the cost of MFCs. Up to now, significant research efforts have been devoted to the exploration of alternative ORR catalysts, and some inexpensive ORR catalysts, such as metal oxide^5^, ^6^, ^7^, ^8^ and nonmetal heteroatom doped carbon,^9^, ^10^, ^11^, ^12^, ^13^, ^14^, ^15^, ^16^ have been developed for the replacing of Pt‐based catalysts. However, it is still challenging to achieve satisfying ORR performance with these inexpensive catalysts due to the sluggish kinetics of ORR. It was shown recently that modification of the nonmetal heteroatom doped carbon with transition metal species could promote its ORR activity to a level comparable to that of Pt‐based catalyst.^17^, ^18^, ^19^, ^20^, ^21^ Although the detailed mechanism for this performance enhancement is not clearly understood yet,^22^, ^23^ these results clearly outlined the potential of the metal and nonmetal heteroatom codoped carbon for the development of high performance and low cost ORR catalyst.

Metal–organic framework (MOF) is an emerging type of highly porous material assembled with metal ions and organic linkers. A spectrum of metal centers and functional organic linkers can be used for the construction of MOFs. Therefore, both the porous properties and chemical properties of MOF can be finely tuned simply by selecting and matching its structural moieties. Owing to its ease of preparation and multifunctional nature, MOF stands as a versatile platform for the development of electrochemically active materials for different energy conversion and storage devices.^24^, ^25^ An important application of MOF is the use of MOF as the precursor for the preparation of metal incorporated porous carbon nanostructures because the uniform arrangement of metal ions in MOF is particularly beneficial for the controlled immobilization of metal in the carbon matrix.^25^, ^26^ So far, the MOF precursor based carbon materials have been used as for the fabrication electrodes for proton exchange membrane fuel cell,^27^ lithium ion battery,^28^ and so on.

Herein, we report the synthesis of a class of Co‐MOF‐derived dual metal and nitrogen codoped carbon (M/CoNC) catalysts through a pyrolysis procedure. The obtained M/CoNC (M: Ni, Fe, Zn, and Cu) catalysts are highly porous with metal and nitrogen uniformly distributed within the graphite carbon matrix. These intriguing features of the prepared M/CoNC catalysts enable high‐density active sites for ORR, regulated pathways for electrical, and active oxygen species, and thus the superior ORR performance in alkaline and neutral media. Particularly, the Ni/CoNC catalysts exhibit the best ORR performance with an onset potential of 0.347 V in phosphate‐buffered solution (PBS) (pH = 7) solution and excellent stability. Furthermore, while using the Ni/CoNC catalyst as air cathode catalyst in an MFC device (denoted as Ni/CoNC–MFC), the as‐fabricated Ni/CoNC–MFC device achieved a remarkable power density of 4335.6 mW m^−2^ (based on the projected surface area of the anode) as well as outstanding durability that can steadily operate for more than 755 h.

The synthetic process of the M/CoNC catalyst is schematically shown in **Figure** **1**a. Highly thermal stable MOF, zeolitic imidazolate framework (ZIF‐67),^29^, ^30^, ^31^ was prepared and used as the carbon precursor to yield the CoNC catalyst. In addition to Co‐MOF, Ni‐loaded Co‐MOF (Ni/Co‐MOF) was also fabricated by mixing the NiCl_2_ solution (Figure 1a) with Co‐MOF solution. Upon loading with Ni ions, the color of the Co‐MOF solution turned blue (inset in Figure 1b). Ni/Co‐MOF powder was obtained by drying and then carbonized to form the Ni/CoNC catalyst. X‐ray diffraction (XRD) pattern of the as‐prepared Co‐MOF agrees well with the simulated one, suggesting the successful formation of porous framework with high crystallinity. Although the stability of most MOF materials in aqueous conditions remains a major concern for their applications, we found that the metal loading process here did not destruct the framework structure of Co‐MOF. All the characteristic peaks from Co‐MOF were present on the XRD spectra of the Ni/Co‐MOF (Figure 1b), clearly demonstrating the preservation of the Co‐MOF structure. The morphology of the carbonized Ni/Co‐MOF (abbreviated as Ni/CoNC catalyst in the following) was first probed with transmission electron microscopy (TEM) measurements. The representative TEM image (Figure 1c) showed that the Ni/CoNC catalyst was composed of the microporous carbon phase and metal nanoparticle enriched phase. The elemental distribution of the Ni/CoNC catalyst was further studied with high angle annular dark‐field scanning transmission electron microscopy (HAADF STEM) measurements. As shown in Figure 1d1–d4,e1–e4, C, N, Ni, and Co are evenly dispersed in the microporous carbon phase. The nanoparticle‐enriched phase, which was closely wrapped by a few layers of graphite carbon and uniformly distributed in the microporous carbon phase (Figure 1d), mainly consisted of Ni, Co, and N, suggesting the alloying between the two metal species and nitrogen. The spatial confinement of the metal species in the Ni/CoNC catalyst is of particularl importance for suppressing the dissolution and aggregation of the metal nanoparticles, which is advantageous for maintaining a high stability under working condition.

**Figure 1:**
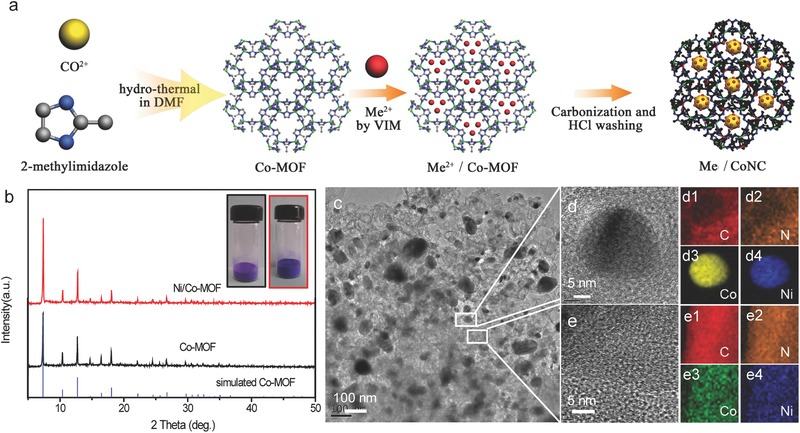
a) Schematic diagram of the preparation process for the metal/CoNC catalysts. b) XRD spectra of the simulated Co‐MOF, as‐prepared Co‐MOF, and Ni/Co‐MOF samples. c–e) TEM images of the as‐synthesized Ni/CoNC catalyst. EELS elemental maps of d1, e1) C, d2, e2) N, d3, e3) Co, and d4, e4) Ni.

To detect the components of the as‐prepared Ni/CoNC catalyst, X‐ray photoelectron spectroscopy (XPS) investigations were carried out. The presence of N was clearly observed in the XPS survey spectra of both the CoNC and Ni/CoNC samples (**Figure** **2**a). Characteristic O 1s peak, the presence of which could be possibly attributed to the environmental oxygen species adsorbed on the Ni/CoNC catalyst, was also observed. No obvious peak from Co and Ni was observed in the survey spectra. This could be because of the encapsulation of Co species within the graphite phase of the Ni/CoNC catalyst and the low Co content in the Ni/CoNC catalyst. High‐resolution XPS scan was further conducted to gain insight into the chemical state of the elements. As can be seen from the high‐resolution C1s spectrum of the Ni/CoNC catalyst, a deconvoluted peak at 286.6 eV (corresponding to C–N) was identified (Figure 2b), confirming the successful doping of nitrogen.^32^ The characteristic peaks for Pyridinic N (398.4 eV), pyrrolic N (399.8 eV), graphite N (400.9 eV), and pyridine N (402.4 eV) were observed in the fitted N 1s peak (Figure 2c).^33^ Co species of different chemical states were also detected from the Co 2p spectra (Figure 2d).^34^ The appearance of Co–N peak provides concrete evidence for the formation of Co–N–C structures. Ni species^35^ were also identified in the Ni/CoNC catalyst (Figure 2e). Raman measurements were performed to characterize the graphite structure of the catalysts. Both the D band (1329 cm^−1^) and G band (1573 cm^−1^) were identified in the Raman spectra of both the CoNC and Ni/CoNC catalysts (Figure S1, Supporting Information). The ratio of the integrated intensity (*I*_D_/*I*_G_) for the Ni/CoNC sample was calculated to be 0.71, which is lower than that of the CoNC sample, implying that the Ni/CoNC sample has higher degree of graphitization.^36^ This observation agrees well with the results from the TEM images that the alloy structure tended to lead to the formation of highly graphitized carbon. Such an improved graphitization is attributed to the nickel‐catalyzed graphitization mechanism.^37^, ^38^ The carbon–carbon bond will be ruptured at first by the catalyst Ni at the interface between the disordered carbon and Ni under high heat treatment temperature. Then, the carbon dissolves in solid or molten Ni and precipitates as graphitic carbon easier than that without Ni catalyst, and thus leading to a higher degree of graphitization in the presence of Ni.^38^

**Figure 2:**
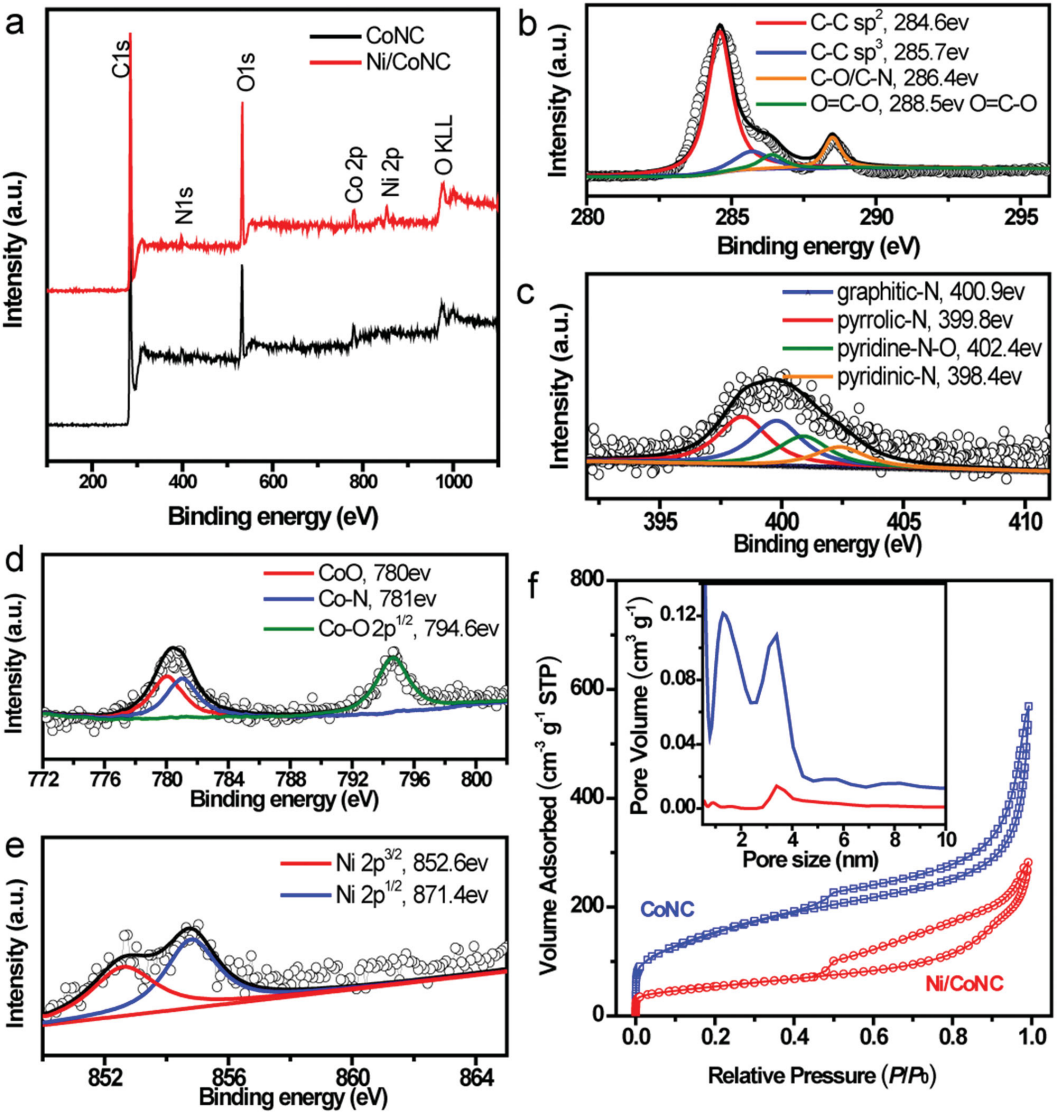
a) XPS survey spectra of the CoNC and Ni/CoNC catalysts. Core level: b) C1s, c) N1s, d) Co 2p, and e) Ni 2p XPS spectra of the Ni/CoNC catalyst. f) Nitrogen isothermal adsorption/desorption curves of the CoNC and Ni/CoNC catalysts. The inset is their corresponding pore size distribution.

Figure 2f shows the typical nitrogen isothermal adsorption/desorption curves of the CoNC and Ni/CoNC catalysts. Significantly, both the prepared CoNC catalyst and Ni/CoNC catalyst exhibited typical type IV N_2_ adsorption isotherm with H1 hysteresis loop. The sharp increase in the volume of adsorbed N_2_ with the pressure increase at around *P*/*P*_0_ = 0 in the N_2_ isotherm of the CoNC catalyst reveals the presence a large amount of micropores in the catalysts. In contrast, the Ni/CoNC catalyst showed much lower micropore content, as suggested from its N_2_ isotherm. Such difference in the pore content implies that the Ni specie occupied most of the micropores in Co‐MOF after loading. This structure arrangement in turn led to the formation of Ni/CoNC structure with uniform composition after carbonization. The average pore size, BET specific area, and pore volume of the CoNC catalyst was determined to be 1.3 nm, 555 m^2^ g^−1^, and 0.88 cm^3^ g^−1^, while 3.4 nm, 194 m^2^ g^−1^, and 0.44 cm^3^ g^−1^ for Ni/CoNC catalyst, respectively.

The ORR activities of the CoNC and Ni/CoNC catalysts were first evaluated in alkaline condition with cyclic voltammetry (CV) measurements in N_2_‐ or O_2_‐saturated alkaline condition. The commercial 20 wt% Pt/C catalyst was also tested as the reference. As depicted in **Figure** **3**a, no characteristic peak was presented in the CV curve of the Ni/CoNC catalyst when saturating the KOH solution with N_2_, demonstrating the good stability of the catalyst in the whole electrochemical window. Well‐developed cathodic peaks were observed for both the CoNC and the Ni/CoNC catalysts in the CV curves when saturating the alkaline solution with O_2_, indicating the pronounced ORR activity of the catalysts. A high cathodic current compared to that of the reference Pt/C was also detected for Ni/CoNC catalyst. The ORR activity of the catalyst was also tested by linear sweep voltammetry (LSV) on a rotating disk electrode in the solution of KOH saturated with O_2_. At a rotation rate of 1600 rpm, the Ni/CoNC electrode showed an onset potential of −0.085 V, which is positively shifted by 134 mV compared to that of CoNC electrode, and even comparable to that of Pt/C electrode. Moreover, the Ni/CoNC electrode was able to achieve a remarkably high diffusion limit current of 6.66 mA cm^−2^, which is similar to that of Pt/C electrode. These present onset and half‐wave potentials are also significantly more positive than the values of recently reported nonnoble‐based catalysts in alkaline electrolyte (Table S1, Supporting Information).^39^, ^40^, ^41^, ^42^ These results demonstrate the excellent ORR performance of the Ni/CoNC catalyst under alkaline condition. The Ni/CoNC catalyst possessed better ORR property than the CoNC catalyst in alkaline solution, which could possibly be associated with the higher degree of graphitization of the Ni/CoNC. To determine the kinetic parameters of the Ni/CoNC catalyst for ORR, LSV measurements were performed at different rotating speeds from 400 to 2000 rpm (Figure S2, Supporting Information). With the increase in the rotating speed, the current density of the polarization curves increased accordingly due to the shortened diffusion distance. The Koutecky–Levich (K–L) equation was used to derive the electron‐transfer number of the catalyst. **Figure** **4**c and Figure S3 in the Supporting Information display the K–L plots of all the samples that were obtained by linear fitting of the inverse square root of rotating speed versus reciprocal current density at different rotating rates. The linear K–L plots reveal that the all the catalysts act up to the first‐order reaction kinetics toward the concentration of O_2_ from −0.3 to −0.7 V. The calculated electron transfer numbers for the Ni/CoNC catalyst is in the range of 3.79–4, which are closed to those of commercial Pt/C catalyst (Figure S3, Supporting Information). This demonstrates that the ORR with the Ni/CoNC catalyst processes a desired four‐electron process.

**Figure 3:**
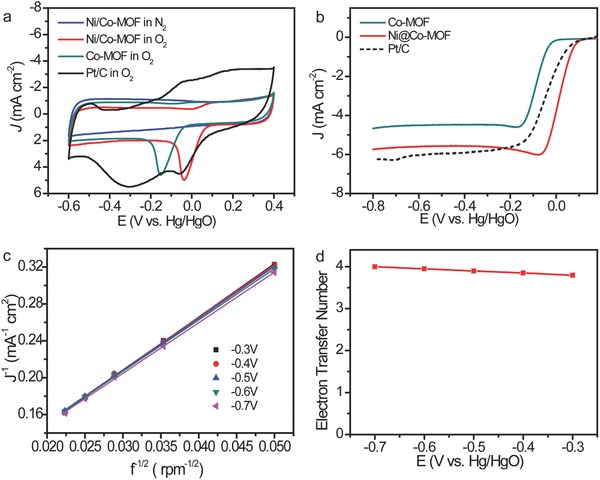
a) CV curves of the CoNC, Ni/CoNC, and Pt/C electrodes in O_2_‐ or N_2_‐saturated 0.1 m KOH solutions. b) LSV curves of the CoNC, Ni/CoNC, and Pt/C electrodes in 0.1 m KOH solution saturated with O_2_ at 1600 rpm. c) K–L plots of the Ni/CoNC electrode at different potentials. d) The calculated ORR electron‐transfer number for the Ni/CoNC catalyst at different potentials.

**Figure 4:**
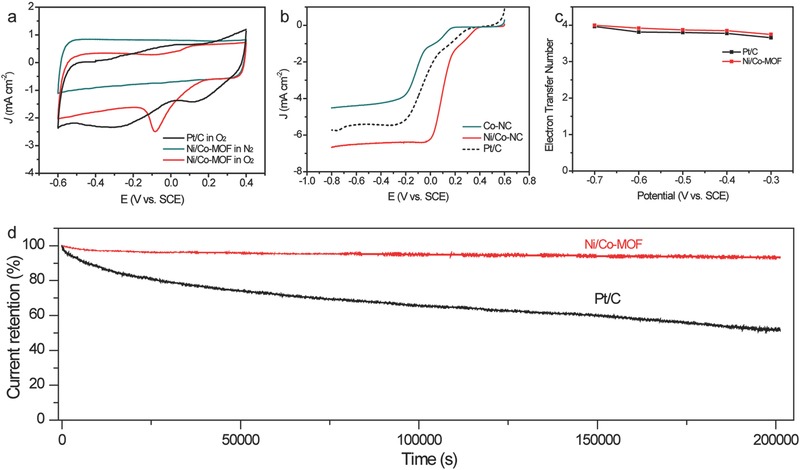
a) CV curves of the Ni/CoNC and Pt/C catalysts in O^2^‐ and Ar‐saturated 0.01 m PBS solution. b) LSV curves of the Ni/CoNC and Pt/C catalysts collected in O_2_‐saturated 0.01 m PBS at 1600 rpm. c) The calculated ORR electron‐transfer number for the Ni/CoNC catalyst at different potentials. d) Current retention curves of the Ni/CoNC and Pt/C catalysts recorded at −0.3 V in O_2_‐saturated 0.01 m PBS electrolyte.

To evaluate the potential of the as‐prepared Ni/CoNC as the cathode catalyst of MFC, the CV measurements were performed in PBS buffer (pH = 7, 0.01 m). Figure 4a presents the CV curves of the Pt/C and Ni/CoNC electrodes in a solution of Ar or O_2_‐saturated PBS solution. The Ni/CoNC catalyst was also electrochemically stable in neutral PBS solution since the shape of its CV curve in N_2_‐saturated PBS solution was fairly rectangular. Well‐defined cathodic peaks corresponding to the oxygen reduction was clearly visible when the PBS buffer was saturated with O_2_, which indicates that the Ni/CoNC catalyst also has good ORR activity in neutral PBS solution. Figure 4b compares the LSV curves of the as‐prepared CoNC, Ni/CoNC, and Pt/C catalysts collected at a rotating speed of 1600 rpm. Remarkably, the Ni/CoNC catalyst exhibited more positive onset potential and high limiting current than those of the Pt/C catalyst, further demonstrating the superior ORR performance of the Ni/CoNC catalyst in neutral aqueous environment. More importantly, these present onset and half‐wave potentials are also comparable to the values of recently reported nonnoble‐based catalysts in alkaline electrolyte (Table S2, Supporting Information).^43^, ^44^, ^45^, ^46^ The stability of the catalysts was surveyed with chronoamperometric measurements at a potential of −0.3 V versus saturated calomel electrode (SCE) in O_2_‐saturated neutral PBS buffer (Figure 4d). Substantial activity decay was clearly observed for the reference Pt/C catalyst during the whole measurement and only 52% of its initial activity was retained after 200 000 s. In contrast, the Ni/CoNC catalyst showed negligible activity loss under identical condition. The catalytic activity retention was as high as 93% for the Ni/CoNC catalyst after 200 000 s. The methanol tolerance of the Ni/CoNC catalyst was further assessed. As evidenced in Figure S4 in the Supporting Information, a sharp decrease in the catalytic activity was found for the Pt/C catalyst upon methanol injection. In comparison, there was only slight performance loss for the Ni/CoNC catalyst with the introduction of methanol. These results clearly demonstrated the outstanding stability of the Ni/CoNC. The good stability of the Ni/CoNC catalyst could be originated from its unique microstructures with metal species closely wrapped with in the graphite carbon.

More importantly, our approach to synthesize the Ni/CoNC catalyst is also applicable for the preparation of other M/CoNC catalysts, which allows us to gain deeper insight into the interplay between the composition of the M/CoNC catalyst and their catalytic activity. Several kinds of M/CoNC catalysts including Cu/CoNC, Zn/CoNC, and Fe/CoNC were readily obtained by simply mixing Co‐MOF powder with different metal chloride solutions. All the Cu/CoNC, Zn/CoNC, and Fe/CoNC catalysts showed homogeneous microstructure as well as uniform elemental distribution (Figures S5–S7, Supporting Information), similar to Ni/CoNC catalyst. The ORR performance of these catalysts in neutral PBS buffer was also studied by LSV measurements (Figure S8, Supporting Information). All these as‐prepared M/CoNC catalysts showed comparable ORR performances to the Pt/C electrode, except for Fe/CoNC catalyst. The onset potential and half‐wave potential of these catalysts are listed in Table S3 in the Supporting Information.

To demonstrate the feasibility of the as‐prepared catalysts as a high‐performance cathodic ORR catalyst for MFCs, we further assembled a single‐chamber MFC device by using the Ni/CoNC catalyst as air cathodic catalyst since it has the highest ORR activity in neutral PBS solution (denoted as Ni/CoNC–MFC; see the Experimental Section for details). *Escherichia coli* cell was used as an electrogenic bacterial strain that generates electrons from organic substrate as previously described.^47^, ^48^ Notably, the MFC performance is difficult to compare directly with other literatures because of the different adopted parameters, such as the cathodic reaction, organic substrate, buffer system, inoculated bacterial strain, cell configuration, etc. Herein, a similar MFC device was also fabricated for better comparison by using commercial 40 wt% Pt/C as air cathode under the same identical configuration and operation conditions (denoted as Pt/C–MFC). The polarization curves and power outputs of the as‐fabricated Pt/C–MFC and Ni/CoNC–MFC devices are presented in **Figure** **5**a. The open‐circuit potential for the Ni/CoNC–MFC device reached about 0.60 V, which is closed to that of Pt/C–MFC (0.61 V). However, in comparison to Pt/C–MFC device, the cell voltage of the Ni/CoNC–MFC device decreased more slowly with the decreasing load resistance, revealing the superior ORR activity and charge transfer rate of the Ni/CoNC–MFC device. Further, the Ni/CoNC–MFC device was able to yield a maximum power density of 4335.6 mW m^−2^ (based on the projected surface area of the anode), which is substantially larger than the value obtained for Pt/C–MFC (2520.8 mW m^−2^) at the same operation conditions. Importantly, the Ni/CoNC–MFC device exhibited an excellent durability with the continuous supply of organic substrates. As displayed in Figure 5b, the continuous voltage generation from the Ni/CoNC–MFC device in three consecutive feeding cycles was more than 755 h. The voltage decreased gradually with the depletion of nutrients in the MFC, and almost recovered to its initial value after the replenishment of fresh anolyte.

**Figure 5:**
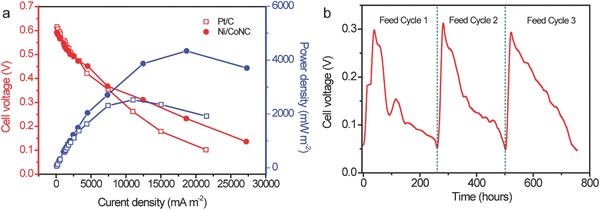
a) Comparisons of polarization curves and power outputs for the Pt/C–MFC and Ni/CoNC–MFC devices. b) Voltage output of the Ni/CoNC–MFC device as a function of operating time with an external load resistance of 500 Ω.

In summary, a series of M/CoNC (M: Ni, Fe, Zn, and Cu) catalysts with high ORR activities in both alkaline and neutral conditions was developed with Co‐MOF. The as‐synthesized M/CoNC (M: Ni, Fe, Zn, and Cu) catalysts showed large surface area and highly uniform composition with N and metal species well encapsulated within the graphite structure. All these M/CoNC catalysts were able to deliver prominent ORR activities, even comparable to commercial Pt/C catalyst. Moreover, a high‐performance and stable MFC device was also demonstrated by using the as‐prepared Ni/CoNC as air cathode catalyst. Significantly, the Ni/CoNC–MFC device exhibited substantially higher power density compared to that of the Pt/C–MFC device. The Ni/CoNC–MFC device reached a maximum power density of 4335.6 mW m^−2^ and excellent durability. Our present work not only shows that the produced M/CoNC catalysts are promising replacements for the Pt‐based ORR catalysts in MFCs, but also indicates that the strategy of using MOF as the precursor for the preparation of functional carbon materials is a versatile method that can be used in a number of applications.

## Experimental Section

*Synthesis of Co*‐*MOF and Metal‐Loaded Co‐MOF*: 0.6 × 10^−3^ m cobalt acetate and 1.2 × 10^−3^ m 2‐methylimidazole were mixed in 45 mL dimethylformamide (DMF). After mixing, the mixture was transferred into a 50 mL Teflon lined autoclave and heated at 130 °C for 24 h. The solid Co‐MOF products were collected and rinsed with DMF for several times, followed by drying at 80 °C. Different metal precursors (FeCl_2_, NiCl_2_, CuCl_2_, and ZnCl_2_) were used as metal precursors for the preparation of the metal‐loaded Co‐MOF by the vacuum‐assisted impregnation method (VIM). In a typical synthesis, 500 mg dried Co‐MOF was treated under vacuum (<10 Pa) to remove impurities and trap air in the microporous structure, and then the Co‐MOF powder was added to a 20 mL FeCl_2_, NiCl_2_, CuCl_2_, or ZnCl_2_ hexane solution (0.005 mol L^−1^) under vacuum. The mixture was further stirred for 3 h to ensure the complete loading of metal species in the Co‐MOF. Finally, the M‐Co‐MOF products were collected and dried first at room temperature and then at 150 °C under vacuum.

*Synthesis of M*/*CoNC Catalysts*: The M/CoNC catalysts were synthesized by direct carbonization of the Co‐MOF or M‐Co‐MOF. The carbonization was performed at 800 °C for 4 h with a ramping rate of 5 °C min^−1^ in an N_2_ atmosphere.

*Characterizations*: XRD spectra were collected on an XRD instrument (D/MAX‐RB RU‐200B, Japan) using a Cu Kα (*λ* = 0.15406 nm) radiation source. The Raman measurements were performed with a Raman spectroscopy (inVia, Renishaw plc, Gloucestershire, UK) with a 514.5 nm laser source. The TEM images, high‐resolution transmission electron microscopy (HRTEM) images, HAADF STEM images, and element mapping images were obtained with a JEM2010‐HR microscopy (FEI Tecnai G2 F30) operating at 300 kV. The porous properties of the samples were probed by nitrogen physisorption at 77 K with an ASAP 2020 V3.03 H instrument and the distribution of the pore size was derived with the Barrett Joyner Halenda (BJH) methods. XPS analysis was conducted on a VG Multilab 2000×‐spectrometer using an Al Kα X‐ray source (1486 eV).

*Electrochemical Measurements*: The electrochemical measurements were performed with an Autolab PGSTAT 30 electrochemical test system (Eco.Chemie B.V, The Netherlands) in a standard three‐electrode cell with a SCE or mercury/mercury oxide electrode as the reference electrode, a Pt plate as the counter electrode, and the catalyst film coated rotating disk electrode as the working electrode. The catalyst ink was prepared by dispersing 5 mg of the catalyst in 1 mL isopropanol contacting 20 μL Nafion solution (5 wt%, DuPont). The catalyst loading was 0.5 mg cm^−2^ for both M/CoNC and 20 wt% Pt/C catalyst. The electrolytes employed were 0.1 m KOH and 0.01 m PBS buffer for the ORR tests in alkaline solution and neutral solution, respectively. ORR tests were conducted on a rotating‐disk electrode system, in which the rotational speed was varied from 400 to 2000 rpm with a scan rate of 10 mV s^−1^. The electrolyte was bubbled with N_2_ or O_2_ for 30 min before the measurement. The ORR stability test was carried out at −0.3 V in O_2_‐saturated 0.01 m PBS electrolyte.

*Construction and Measurements of MFC*: An air‐cathode single‐chamber cube MFC was constructed, consisting of a anode chamber (5 cm × 5 cm × 4 cm) and the membrane cathode assemble (MCA) on one side (5 cm × 5 cm). Herein, carbon cloth (AvCarb 1071 HCB, Fuel Cell Earth) of 2.5 cm × 2.5 cm was used as anode, and carbon paper (Shanghai Hesen, China) of 5 cm × 5 cm was used as the air cathode. The air cathode was prepared by coating a paste on carbon paper to produce a uniform film with a catalyst effective area of 16 cm^2^. The paste was prepared by mixing 7 μL mg^−1^ 5% Nafion solution (DuPont), 0.1 mL ethanol, and the Ni/CoNC catalyst or 40 wt% Pt/C catalyst (Shanghai Hesen, China). Finally, the as‐synthesized air cathode was dried at room temperature for 24 h and then hot‐pressed on one side of a cation exchange membrane (CEM) to form an MCA. The mass loading of Ni/CoNC and 40 wt% Pt/C catalysts was about 0.5 mg cm^−2^.

*E. coli* K‐12 (strain) were cultured aerobically at 37 °C for 10 h in a standard Luria–Bertani (LB) medium consisting of 10.0 g of peptone, 5.0 g of NaCl, and 3.0 g of beef powder per liter. For the MFC experiments, 2 mL of the overnight *E. coli* K‐12 culture was inoculated to 18 mL anolyte (saturated with nitrogen for 20 min before inoculation). The anolyte was PBS (10.00 g L^−1^ of NaHCO_3_ and 11.20 g L^−1^ of NaH_2_PO_4_ 2 H_2_O) containing 5 × 10^−3^ m of 2‐hydroxy‐1,4‐naphthoquinone (HNQ, Sigma‐Aldrich), 10.00 g L^−1^ of glucose, and 5.00 g L^−1^ of yeast extract. The polarization curves and power outputs were measured about 6 h after inoculation. The MFC cell voltages of a certain external resistance (*R*_ex_), which varied over a range from 10 kΩ to 10 Ω, were recorded. The power density (W m^−2^) was calculated according to *P* = *IU*/*A*, where *I* (*A*) is the current, *U* (*V*) is the voltage and *A* (m^2^) is the project area of the anode. The external load resistor for the batch‐fed mode experiments was 1 kΩ, and 80% of the anodic medium is replaced by the fresh anodic solution when the voltage dropped to 50 mV, indicating the depletion of glucose.

## Supporting information

As a service to our authors and readers, this journal provides supporting information supplied by the authors. Such materials are peer reviewed and may be re‐organized for online delivery, but are not copy‐edited or typeset. Technical support issues arising from supporting information (other than missing files) should be addressed to the authors.

Supplementary
