# Supplementary material for: Metal–Organic‐Framework‐Derived Dual Metal‐ and Nitrogen‐Doped Carbon as Efficient and Robust Oxygen Reduction Reaction Catalysts for Microbial Fuel Cells
Source: Adv Sci (Weinh). 2015 Dec 3;3(2):1500265. doi: 10.1002/advs.201500265 (PMC5063114; doi:10.1002/advs.201500265)
Supplement: Supplementary file 1 — Supplementary [file ADVS-3-0f-s001.pdf]

Copyright WILEY-VCH Verlag GmbH & Co. KGaA, 69469 Weinheim, Germany, 2015.

Supporting Information

for *Adv. Sci.*, DOI: 10.1002/advs.201500186

Metal–Organic-Framework-Derived Dual Metal- and Nitrogen-Doped Carbon as Efficient and Robust Oxygen Reduction Reaction Catalysts for Microbial Fuel Cells

Haolin Tang, Shichang Cai, Shilei Xie, Zhengbang Wang, Yexiang Tong, Mu Pan, and Xihong Lu**

Copyright WILEY-VCH Verlag GmbH & Co. KGaA, 69469 Weinheim, Germany, 2013.

Supporting Information

Metal Organic Framework Derived Dual Metal and Nitrogen doped Carbon as Efficient and Robust Oxygen Reduction Reaction Catalysts for Microbial Fuel Cells

Haolin Tang, [a] Shichang Cai,[a] Shilei Xie,[b] Zhengbang Wang,[c] Yexiang Tong,[b] Mu Pan,[a] and Xihong Lu[b]**

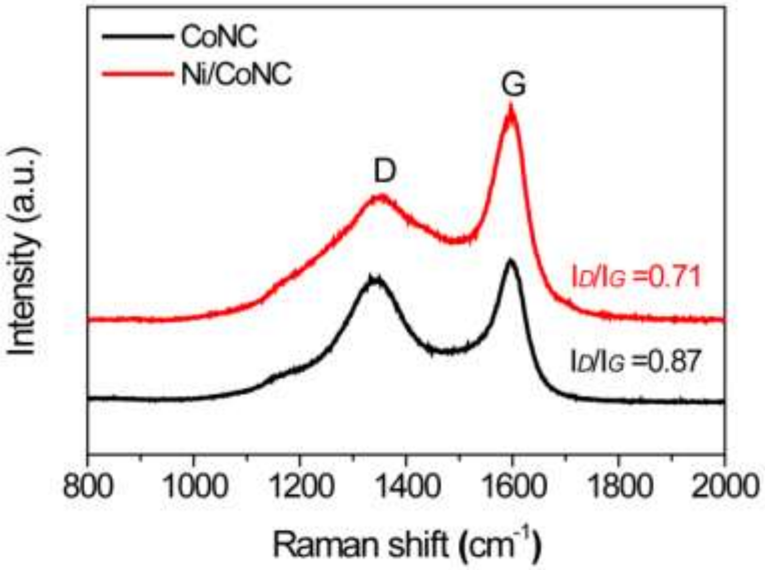

Figure S1. Raman spectra of the CoNC fand Ni/CoNC catalysts.

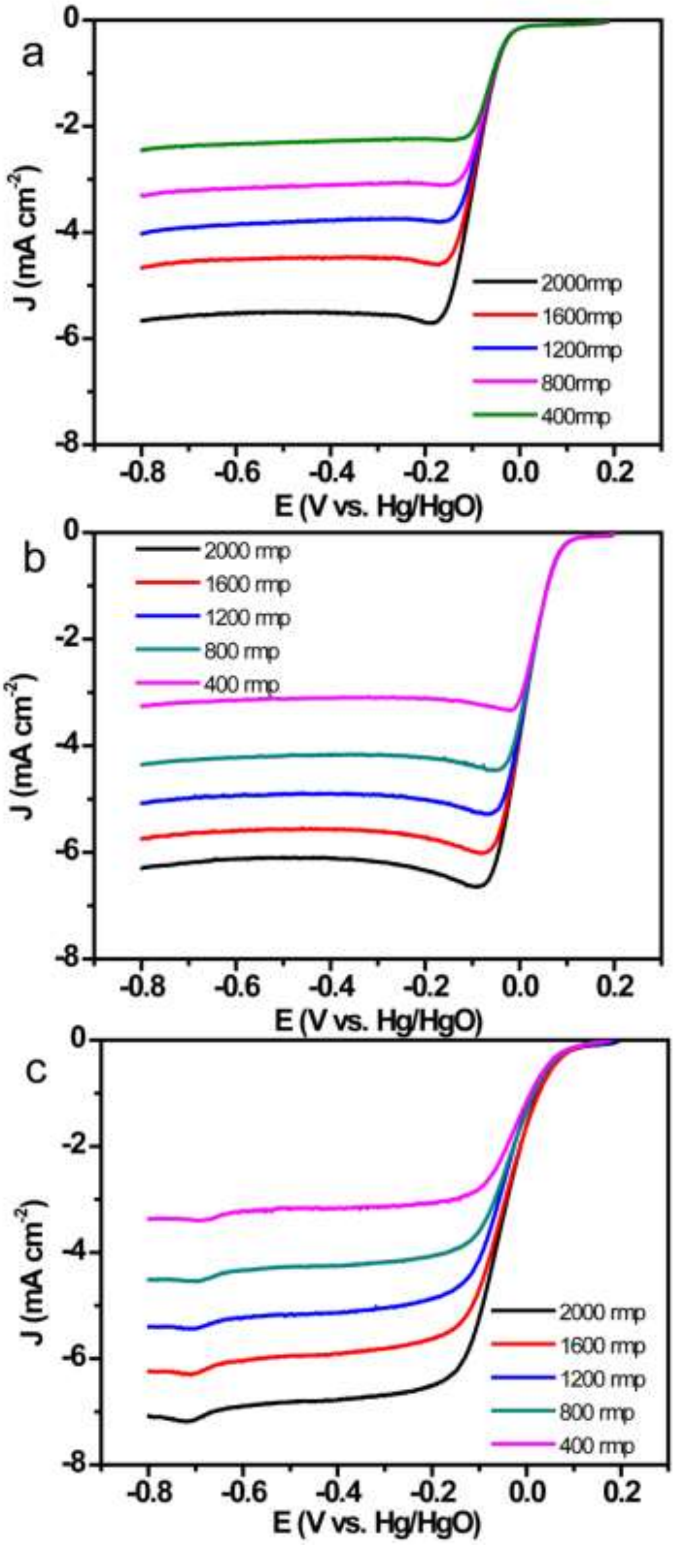

Figure S2. LSV curves of (a) CoNC catalyst, (b) CoNC catalyst and (c) Pt/C catalyst in 0.1M KOH solution saturated with O_2 at different rotating speeds.

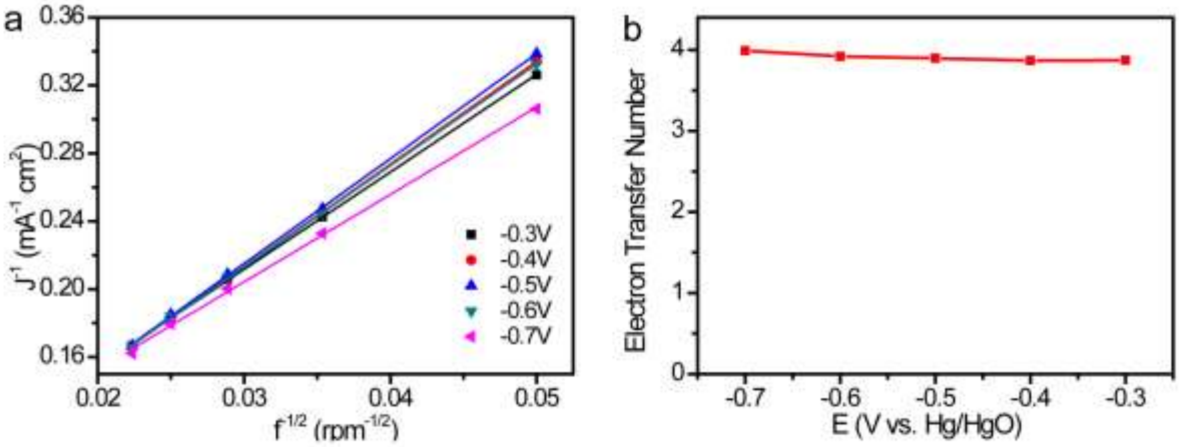

Figure S3. (a) Koutecky–Levich (K-L) polts of the Pt/C electrode at different potentials. (d) The calculated ORR electron-transfer number for the Pt/C catalyst at different potentials.

Table S1. Summary of ORR parameters of various electrocatalysts in alkaline media

	$J_{@-0.6V}$ (mA cm^{-2})	E_{ocp} (V vs. Hg/HgO)	$E_{1/2}$ (V vs. Hg/HgO)	n	ref
**Ni/CoNC**	**6.24**	**0.070**	**-0.049**	**3.94-4**	**Our work**
20% Pt/C	5.74	-0.033	0.018	3.87-3.99	Our work
nitrogen-doped carbon nanofibers	6.1	0.12 Ag/AgCl)	-0.22	3.8	2
Fe/N doped C	~5.1 (1500r)	0.20	0.03		4
N doped graphene	0.48	-0.04	-0.35		3
N-Fe/Fe_3C@C	6.4	0.21	0.14	3.98	1
N-P codoped porous carbon foams	4.9	0.19	-0.11	3.7-3.8	5
PEDOT hollow spheres	2.5/1400r	~0.1	-0.27	3.11	8
S doped RGO	4.7	~0.16	-0.12	3-3.5	6
Fe-N co-doped Carbon	5.44	0.197	-0.17	3.86	7

[1] Z. Wen, S. Ci, F. Zhang, X. Feng, S. Cui, S. Mao, S. Luo, Z. He, J. Chen, *Adv. Mater.* **2012**, *24*, 1399.

[2] X. Yang, W. Zou, Y. Su, Y. Zhu, H. Jiang, J. Shen, C. Li, *J. Power Sources* **2014**, *266*, 36.

[3] L. Feng, Y. Chen, L. Chen, *ACS Nano* **2011**, *5*, 9611.

[4] L. Birry, P. Mehta, F. Jaouen, J.-P. Dodelet, S. Guiot, B. Tartakovsky, *Electrochim. Acta* **2011**, *56*, 1505.

[5] H. Jiang, Y. Zhu, Q. Feng, Y. Su, X. Yang, C. Li, *Chem. Eur. J.* **2014**, *20*, 3106.

[6] M. Seredych, J.-C. Idrobo, T. J. Bandosz, *J. Mater. Chem. A* **2013**, *1*, 7059.

[7] Y. Su, H. Jiang, Y. Zhu, W. Zou, X. Yang, J. Chen, C. Li, *J. Power Sources* **2014**, *265*, 246.

[8] Z. Guo, Y. Qiao, H. Liu, C. Ding, Y. Zhu, M. Wan, L. Jiang, *J. Mater. Chem.* **2012**, *22*, 17153.

Table S2. Summary of ORR parameters of various electrocatalysts in neutral media

	$J_{@-0.6V}$ (mA cm^{-2})	E_{ocp} (V vs. SCE)	n	ref	c (PBS)
**Ni/CoNC**	**6.66**	**0.347**	**3.92**	**Our work**	**0.01**
20% Pt/C	5.72	0.282	3.81	Our work	0.01
acidic/basic-N-activated carbon	7.18	0.18	4.09	9	0.1 M
Co-PDAP	~6 (1500 rpm)	0.082	3.96	10	---
Fe-C-N	6.1 (2500 rpm)	0.2	3.89	11	---
FeCo- melamine-formaldehyde resin	5.0 (1500 rpm)	0.88 (RHE)	3.96	12	0.2 M

[9] B. Zhang, Z. Wen, S. Ci, S. Mao, J. Chen, Z. He, *ACS Appl. Mater. Interfaces* 2014, 6, 7464.

[10] Y. Zhao, K. Watanabe, K. Hashimoto, *J. Am. Chem. Soc.* 2012, 134, 19528.

[11] Z.-Y. Yang, Y.-X. Zhang, L. Jing, Y.-F. Zhao, Y.-M. Yan, K.-N. Sun, *J. Mater. Chem. A* 2014, 2, 2623.

[12] Y. Zhao, K. Watanabe, K. Hashimoto, *J. Mater. Chem. A* 2013, 1, 1450.

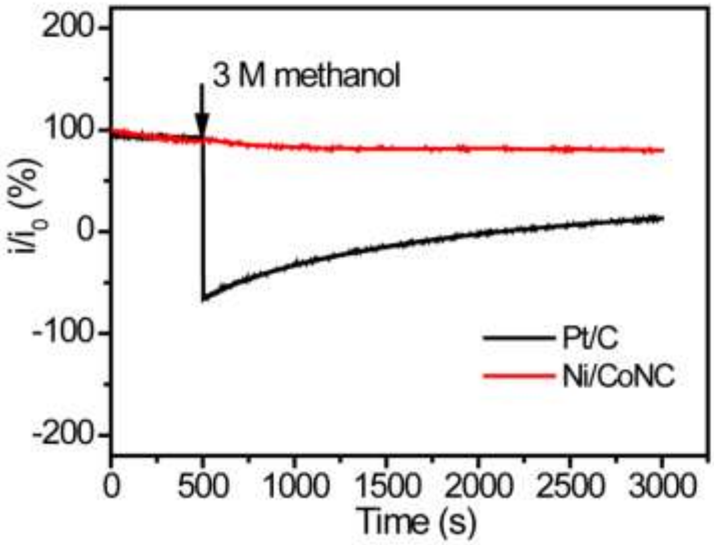

Figure S4. tolerance to methanol of the Ni/CoNC and Pt/C electrodes recorded at -0.3 V in O_2-saturated 10 mM PBS electrolyte with a rotation speed of 1600 rpm.

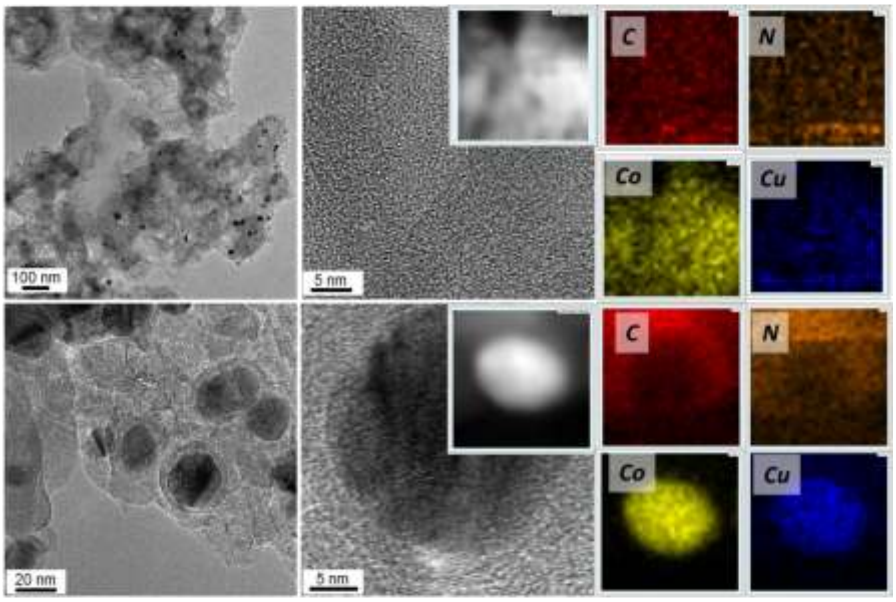

Figure S5. TEM, HRTEM and EELS element mapping images of the as-prepared Cu/CoNC catalyst.

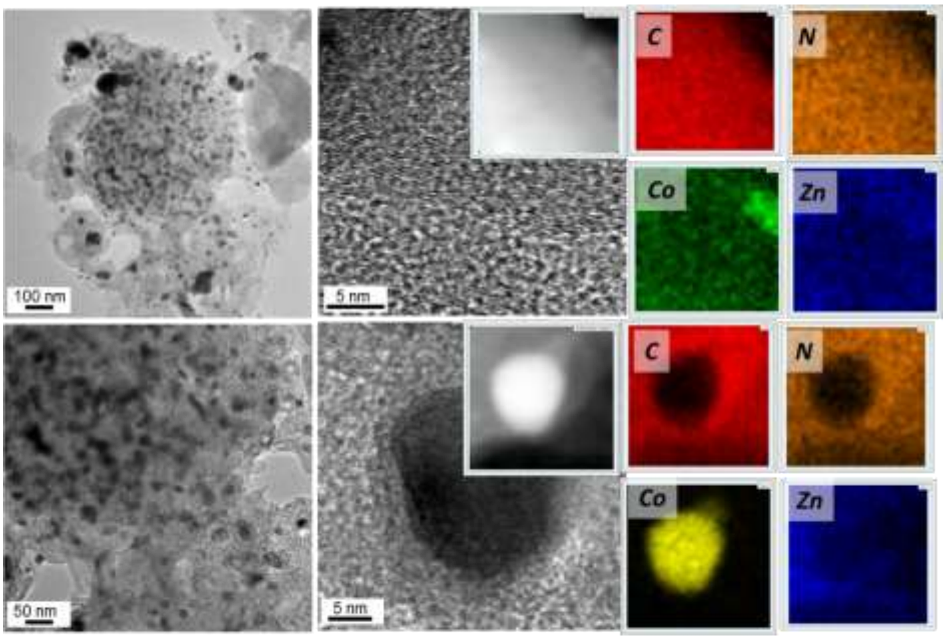

Figure S6. TEM, HRTEM and EELS element mapping images of the as-prepared Zn/CoNC catalyst.

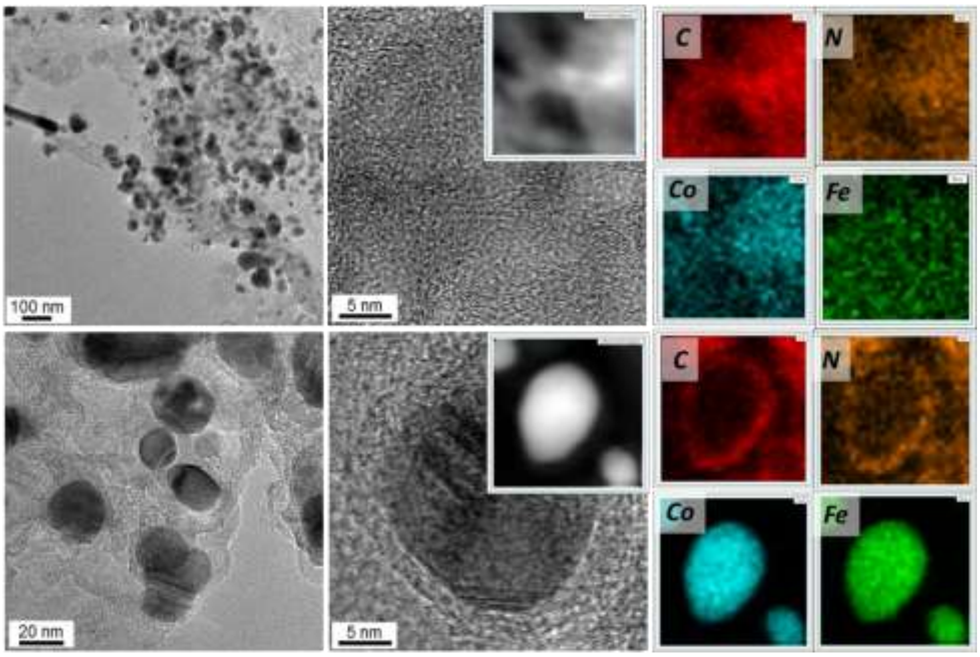

Figure S7. TEM, HRTEM and EELS element mapping images of the as-prepared Fe/CoNC catalyst.

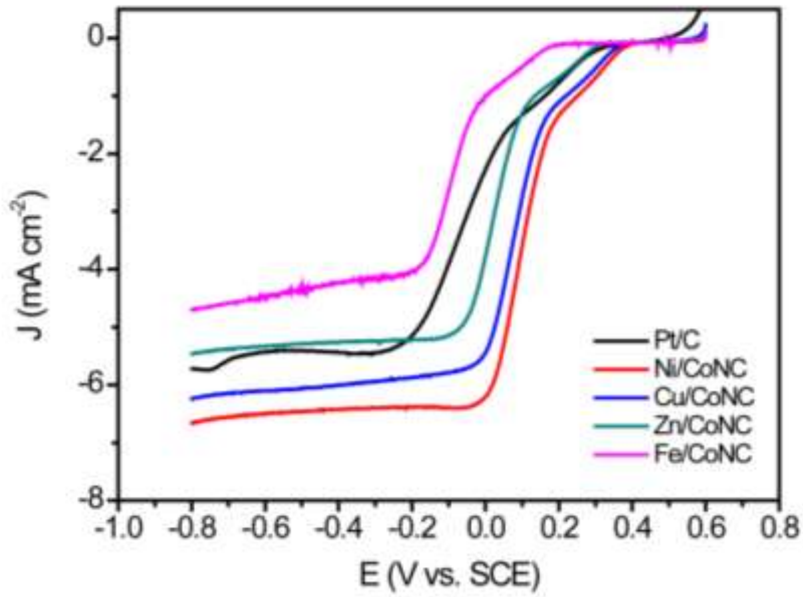

Figure S8. LSV curves of the Pt/C, Zn/CoNC, Cu/CoNC, Fe/CoNC and Ni/CoNC catalysts collected in O_2 –saturated 0.01 M PBS at 1600 rpm

Table S3. The onset potential and half-wave potential of these catalysts in PBS solution

	$J_{@-0.6V}$ (mA cm^{-2})	E_{ocp} (V vs. SCE)	$E_{1/2}$ (V vs. SCE)
Pt/C	5.72	0.282	-0.039
Fe/CoNC	4.70	0.152	-0.088
Zn/CoNC	5.46	0.275	0.034
Cu/CoNC	6.24	0.33	0.088
Ni/CoNC	6.66	0.347	0.108
